# Rosmarinic Acid Activates AMPK to Inhibit Metastasis of Colorectal Cancer

**DOI:** 10.3389/fphar.2018.00068

**Published:** 2018-02-05

**Authors:** Yo-Han Han, Ji-Ye Kee, Seung-Heon Hong

**Affiliations:** Department of Oriental Pharmacy, College of Pharmacy, Wonkwang-Oriental Medicines Research Institute, Wonkwang University, Iksan, South Korea

**Keywords:** rosmarinic acid, colorectal cancer, metastasis, EMT, matrix metalloproteinase, AMPK

## Abstract

Rosmarinic acid (RA) has been used as an anti-inflammatory, anti-diabetic, and anti-cancer agent. Although RA has also been shown to exert an anti-metastatic effect, the mechanism of this effect has not been reported to be associated with AMP-activated protein kinase (AMPK). The aim of this study was to elucidate whether RA could inhibit the metastatic properties of colorectal cancer (CRC) cells via the phosphorylation of AMPK. RA inhibited the proliferation of CRC cells through the induction of cell cycle arrest and apoptosis. In several metastatic phenotypes of CRC cells, RA regulated epithelial–mesenchymal transition (EMT) through the upregulation of an epithelial marker, E-cadherin, and the downregulation of the mesenchymal markers, N-cadherin, snail, twist, vimentin, and slug. Invasion and migration of CRC cells were inhibited and expressions of matrix metalloproteinase (MMP)-2 and MMP-9 were decreased by RA treatment. Adhesion and adhesion molecules such as ICAM-1 and integrin β1 expressions were also reduced by RA treatment. In particular, the effects of RA on EMT and MMPs expressions were due to the activation of AMPK. Moreover, RA inhibited lung metastasis of CRC cells by activating AMPK in mouse model. Collectively, these results proved that RA could be potential therapeutic agent against metastasis of CRC.

## Introduction

Cancer is one of many lethal diseases and shows an imbalance between cell growth and cell death. Colorectal cancer (CRC) is one of the most commonly diagnosed cancers in the world, and approximately half of the patients with CRC exhibit metastatic properties. Metastasis, the spread of cancer to other remote organs, occurs through a complex process, which includes epithelial–mesenchymal transition (EMT), cellular adhesion, migration, and invasion (Wang et al., 2014; Feng et al., 2017). At the beginning of metastasis, cancer cells with epithelial traits are spindle shaped and have lost cell–cell junctions. Subsequently, the cancer cells acquire mesenchymal traits, which promote the migration and invasion of cancer cells and circulate through the blood vessels and lymphatic vessels. Finally, circulating cancer cells adhere to the extracellular matrix of secondary cancer sites (Nicolson, 1991; Li et al., 2009).

Advanced CRC mainly spreads to the liver and the lungs; as the current therapeutic approaches, including surgery, chemotherapy, and irradiation, have not been sufficiently effective, many patients with metastatic cancer die (Fidler, 2003; Geng et al., 2017). In particular, when metastasis occurred at remote sites, the 5-year survival rate declined from 90.3 to 12.5% (Siegel et al., 2014). Although various therapeutic strategies have been achieved in cancer-related science and technology, the therapeutic effects of the current treatments are insufficient (Chen et al., 2017). In addition, the treatments are accompanied by significant side effects, such as intestinal mucositis, neuropathic pain, neutropenia, and diarrhea (Hsieh et al., 2016; Yeh et al., 2016; de Alencar et al., 2017). 5-Fluorouracil (5-FU) and capecitabine, which are widely used to treat CRC, result in undesirable side effects. Several studies have been reported that these anti-cancer agents caused myocardial ischemia and cardiotoxicity (Polk et al., 2013, 2014). Even though many new drugs have been developed to treat CRC, they often showed the unexpected side effects such as genetic alteration and mutation (Aung et al., 2017). To reduce or replace synthetic drugs, natural product or natural product-derived compounds have attracted attention (Afrin et al., 2016; Mayzlish-Gati et al., 2017).

Rosmarinic acid (RA) is an abundant phenolic ester found in *Prunella vulgaris*, which has been used as an oriental medicine (Zimmermann et al., 2011; Chen et al., 2012). Many studies have reported a range of biological effects for *P. vulgaris* and RA, such as anti-inflammatory, anti-diabetes, and anti-cancer activities (Amoah et al., 2016). Recent studies have also elucidated the anti-metastatic effect of RA: Xu et al. (2010a) reported that RA inhibited bone metastasis induced by breast cancer, which was supported in a further study (Xu et al., 2010b), despite the absence of a specific mechanistic study to describe how RA inhibits metastasis.

AMP-activated protein kinase (AMPK) is a heterotrimeric complex that consists of α, β, and γ subunits (Garcia and Shaw, 2017). Numerous roles of AMPK have been reported and the activation of AMPK exerts therapeutic effects in metabolic syndrome, inflammation, and cancer (Li W. et al., 2015; Han et al., 2016). In various cancer cells, the activation of AMPK stimulated the tumor suppressor gene p53, which has been reported to control apoptosis and the cell cycle (Rattan et al., 2005). As activators of AMPK that were not anti-cancer drugs have shown anti-cancer effects in a clinical setting, we investigated whether natural products that could induce the activation of AMPK in cancer cells might yield meaningful anti-cancer activity (Li et al., 2017). We hypothesized that the activation of AMPK was closely related to the RA-mediated anti-metastatic effects on CRC cells and confirmed the effects of RA on metastatic CRC cells and the AMPK-related mechanisms.

## Materials and Methods

### Reagents and Antibodies

Antibodies against caspase-3, caspase-8, caspase-9, Bcl-xL, phospho-AMPK, AMPK, cyclin D1 and CDK4 were obtained from Cell Signaling Technology, Inc. (Danvers, MA, United States). The antibody to Bcl-2 and RA were purchased from Santa Cruz Biotechnology, Inc. (Santa Cruz, CA, United States). The antibody to GAPDH was obtained from Enzo Life Sciences (Farmingdale, NY, United States), and compound C (CC) was obtained from MedChem Express (Monmouth Junction, NJ, United States).

### Cell Culture

Murine colon carcinoma colon 26 (CT26) cells were cultured in Dulbecco’s Modified Eagle’s Medium (DMEM; Thermo Fisher Scientific, Waltham, MA, United States). Human colon carcinoma cell line HCT116 cells were cultured in RPMI1640 (Thermo Fisher Scientific, Waltham, MA, United States). These media were supplemented with 10% fetal bovine serum (FBS; Thermo Fisher Scientific, Waltham, MA, United States) and 1% penicillin–streptomycin (Thermo Fisher Scientific, Waltham, MA, United States) at 37°C in a 5% CO_2_ incubator.

### Cell Viability

Cell proliferation was calculated by using the cell proliferation reagent MTS Kit (Promega Corporation, Madison, WI, United States), as recommended by the manufacturer. Briefly, cells were seeded in 96-well microplates at 3 × 10^3^ cells/well and medium containing various concentrations of RA was added to the wells. After incubation for 24–96 h, the medium was replaced with MTS solution and the absorbance of each well was measured at 490 nm.

### Cell Cycle Analysis

The cell cycle phase was determined by using Muse Cell Cycle Kit in accordance with the manufacturer’s instructions (Millipore, Billerica, MA, United States). Briefly, the RA treated cells (1 × 10^6^ cells/mL) were fixed in 70% fresh ethanol at -20°C for a minimum of 3 h. After the cells were washed in PBS, 200 μL of Cell Cycle Reagent was added and reacted in the dark at room temperature for 30 min. The cells were analyzed by Muse Cell Analyzer and the Muse analysis software (Millipore, Billerica, MA, United States).

### Real-Time RT-PCR

Total RNA was isolated from cells by using QIAzol lysis reagent (QIAGEN, Valencia, CA, United States) in accordance with the manufacturer’s instructions. First-strand cDNA was prepared from an RNA template (2 μg) using the Power cDNA Synthesis Kit (Applied Biosystems, Carlsbad, CA, United States). Reverse transcription was performed at 37°C for 60 min and then at 95°C for 5 min. Real-time RT-PCR was performed by using the Power SYBR^®^ Green PCR Master Mix and StepOne Plus^TM^ Real-time PCR System (Applied Biosystems, Carlsbad, CA, United States). All data were normalized to GAPDH mRNA or β-actin. The sequences of the primers are summarized in **Tables 1**, **2**.

**Table 1:** Sequences for real-time RT-PCR mouse primers.

Gene	Forward (5′–3′)	Reverse (5′–3′)
E-cadherin	AATGGCGGCAATGCAATCCCAAGA	TGCCACAGACCGATTGTGGAGATA
N-cadherin	TGGAGAACCCCATTGACATT	TGATCCCTCAGGAACTGTCC
Snail	TCCAAACCCACTCGGATGTGAAGA	TTGGTGCTTGTGGAGCAAGGACAT
Twist	AGCTACGCCTTCTCCGTCT	TCCTTCTCTGGAAACAATGACA
Vimentin	CGGAAAGTGGAATCCTTGCA	CACATCGATCTGGACATGCTG
Slug	CATTGCCTTGTGTCTGCA	AGAAAGGCTTTTCCCCAGTG
MMP-2	CCCCATGAAGCCTTGTTTACC	TTGTAGGAGGTGCCCTGGAA
MMP-9	AGACCAAGGGTACAGCCTGTTC	GGCACGCTGGAATGATCTAAG
ICAM-1	AACAGAATGGTAGACAGCA	TCCACCGAGTCCTCTTAG
Integrin β1	GGTTTCACTTTGCTGGAGATGG	CAGTTTCTGGACAAGGTGAGCAATA
Cyclin D1	TAGGCCCTCAGCCTCACTC	CCACCCCTGGGATAAAGCAC
CDK 4	AGAGCTCTTAGCCGAGCGTA	TTCAGCCACGGGTTCATATC
NLRC3	GTCAGCTGCTACAAGTCCGGGAC	GAGCCTCAGAGTGCTTCGGTATCC
GAPDH	GACATGCCGCCTGGAGAAAC	AGCCCAGGATGCCCTTTAGT


**Table 2:** Sequences for real-time RT-PCR human primers.

Gene	Forward (5′–3′)	Reverse (5′–3′)
E-cadherin	GTCAGTTCAGACTCCAGCCC	AAATTCACTCTGCCCAGGACG
N-cadherin	CTCCATGTGCCGGATGAC	CGATTTCACCAGAAGCCTCTAC
Snail	ACCACTATGCCGCGCTCTT	GGTCGTAGGGCTGCTGGAA
Twist	AGCTACGCCTTCTCGGTCT	CCTTCTCTGGAAACAATGACATC
Vimentin	TCTACGAGGAGGAGATGCGG	GGTCAAGACGTGCCAGAGAC
Slug	TGTTGCAGTGAGGGCAAGAA	GACCCTGGTTGCTTCAAGGA
MMP-2	AGAAGGCTGTGTTCTTTGCAG	AGGCTGGTCAGTGGCTTG
MMP-9	GAACCAATCTCACCGACGGG	GCCACCCGAGTGTAACCATA
ICAM-1	ATGCCCAGACATCTGTGTCC	GGGGTCTCTATGCCCAACAA
Integrin β1	AACAGAATGGTAGACAGCAT	TCCACCGAGTCCTCTTAG
Cyclin D1	ATGCCAACCTCCTCAACGAC	GGCTCTTTTTCACGGGCTCC
CDK 4	GTGCAGTCGGTGGTACCTG	TTCGCTTGTGTGGGTTAAAA
NLRC3	ATGAGGAAGCAAGAGGTGCGGACGGGC	TCACATTTCAACAGTGCACGTGGGAGC
β-actin	AGAGCTACGAGCTGCCTGAC	CGTGGATGACACAGGACT


### Annexin V Assay

The annexin V assay was conducted by using the Muse Annexin V and dead cell kit (Millipore, Billerica, MA, United States) in accordance with the manufacturer’s instructions. Briefly, the RA-treated cells were collected in culture medium (1 × 10^6^ cells/mL). Then, 100 μL of cell-containing medium was reacted with 100 μL of Muse Annexin V and dead cell reagent in the dark at room temperature for 20 min. The apoptotic cells were measured by using the Muse Cell Analyzer and Muse analysis software (Millipore, Billerica, MA, United States).

### Western Blot Analysis

HCT116 cells and CT26 (1 × 10^6^ cells/well) were cultured with RA for 24 and 48 h, respectively. The treated cells were washed with PBS and lysed in lysis buffer (iNtRon Biotech, Seoul, South Korea) for 1 h. The cell lysates were centrifuged for 15 min and the quantity of protein was evaluated by using a bicinchoninic acid protein assay. The cell lysates were mixed with 2× sample buffer, loaded onto an SDS-polyacrylamide gel, electrophoresed, transferred to a PVDF membrane (Millipore, Billerica, MA, United States). The membranes were divided to target proteins and blocked by 5% BSA/TBST for 2 h, incubated with primary antibodies overnight at 4°C, and then incubated with appropriate secondary antibodies (horseradish peroxidase-conjugated anti-rabbit or anti-mouse immunoglobulin G (Dako, Glostrup, Denmark). The secondary antibodies were detected by the application of ECL solution to the membrane (Santa Cruz, CA, United States). Images were obtained by using the FluorChem E system (ProteinSimple, San Jose, CA, United States).

### Wound Healing Assay

CT26 cells and HCT116 cells were seeded into a 6-well plate (2 × 10^5^ cells/well) and allowed to adhere. A wound area was made by using a 200 μL pipette tip. After the detached cells were removed, the scratched area was maintained in serum-free medium that contained different concentrations of RA (0 and 200 μM) and observed at regular intervals between 0 and 48 h. Randomly selected areas were photographed by using a phase contrast microscope (Leica, Wetzlar, Germany) and wound area was calculated by ImageJ software.

### Invasion Assay

The invasion assay was conducted by using BD BioCoat GFR Matrigel invasion chambers (BD Biosciences, San Jose, CA, United States). CT26 and HCT116 cells (5 × 10^4^ cells) were cultured in serum-free DMEM with RA in the upper chamber and 10% FBS in DMEM in the lower chamber. After incubation, the membrane inserts were washed twice with PBS and fixed in 3.7% paraformaldehyde in PBS for 5 min. The fixed cells were washed by PBS, incubated with 100% methanol for 20 min, and stained with Giemsa for 15 min. The inner sides of the chamber were wiped with a cotton swab and the cells were observed by using a microscope (Leica, Wetzlar, Germany) and cells were counted in random areas per insert.

### Adhesion Assay

To conduct the adhesion assay, CT26 and HCT116 cells (3 × 10^3^ cells) were seeded in pre-coated Matrigel 96-well plates and treated with RA for 24 or 48 h. After incubation, the adherent cells were fixed in 10% formaldehyde for 15 min and stained with 0.05% crystal violet solution for 10 min (Sigma Chemicals, St. Louis, MO, United States). After staining, the crystal violet solution was removed and the plate was washed by PBS. After the plate was completely dried, adherent cells were observed and counted by using a microscope (Leica, Wetzlar, Germany).

### Zymography

The cells were incubated in 6-well plates (3 × 10^5^ cells/well) and treated with RA. The culture media from the wells were diluted 1:1 in zymography sample buffer and subjected to electrophoresis on an 8% SDS-PAGE gel containing gelatin. The electrophoresed proteins were renatured by renaturing buffer (2.5% Triton X-100 in distilled water, pH 7.5) for 30 min at room temperature and then maintained in developing buffer (50 mM Tris-HCl pH 7.5, 10 mM CaCl_2_, and 150 mM NaCl) overnight at 37°C. The activity of MMPs was determined by staining with Coomassie Blue R-250 solution for 30 min, which was detected as white bands. Band intensities were measured using ImageJ software.

### Immunofluorescence Staining

Cells (3 × 10^3^ cells/well) were seeded in an 8-well chamber slide and treated with RA. After incubation, the cells were fixed in 3.7% formaldehyde for 15 min at room temperature and washed with PBS for 5 min. The cells were permeabilized with 0.1% Triton X-100 in PBS for 10 min and then maintained in blocking buffer (3% BSA and 0.1% Triton X-100 in PBS) for 1 h. After blocking, the 8-well chamber slide was reacted with primary antibodies overnight at 4°C in a dark room. The primary antibodies were detected with Alexa 488- and Alexa 568-conjugated secondary antibodies (Thermo Fisher Scientific, Waltham, MA, United States). The slides were washed with PBS, the nuclei were counterstained with DAPI (Sigma Chemicals, St. Louis, MO, United States), and the slides were analyzed by using a Zeiss Observer A1 microscope (Carl Zeiss, Oberkochen, Germany).

### Experimental Lung Metastasis Model

BALB/c female mice (4 weeks, 16–18 g) were purchased from Samtako (Osan, Korea) and mice were divided into three groups (*n* = 7), which were control group, RA-treated group and CC + RA-treated group. RA was dissolved in 0.3% CMC water. To inducing lung metastasis, BALB/c mice were inoculated with CT26 (1 × 10^5^ cells) in 200 μL of PBS via the lateral tail vein. RA-treated group and CC + RA-treated group were administrated with an oral injection of RA (100 mg/kg/day) every day for 14 days. CC was dissolved in 50% dimethyl sulfoxide (DMSO) and it was administered by intraperitoneal injection every 3 days until sacrifice. Control group mice were administered with the same volume of water. The mice were sacrificed at 14 days, lung weights were measured to evaluate tumor metastasis. Tumor colonies in the lungs were counted after the lungs were fixed in Bouin’s solution (Sigma, St. Louis, MO, United States). Separated serum was assayed by the Seoul Medical Science Institute (Seoul Clinical Laboratories, Seoul, South Korea). The research was conducted in accordance with internationally accepted principles for laboratory animal use and care, as stated in Wonkwang University guidelines (WKU17-52).

### Statistical Analyses

The results are expressed as the mean ± standard deviation (SD) of three experiments data, and statistical analyses were performed by using Student’s *t*-test. All statistical analyses were computed by SPSS statistical analysis software version 11.5 (SPSS Inc., Chicago, IL, United States). Values of *p* < 0.05 were considered to represent significant differences.

## Results

### RA Inhibits Proliferation of CRC Cells

**FIGURE 1:**
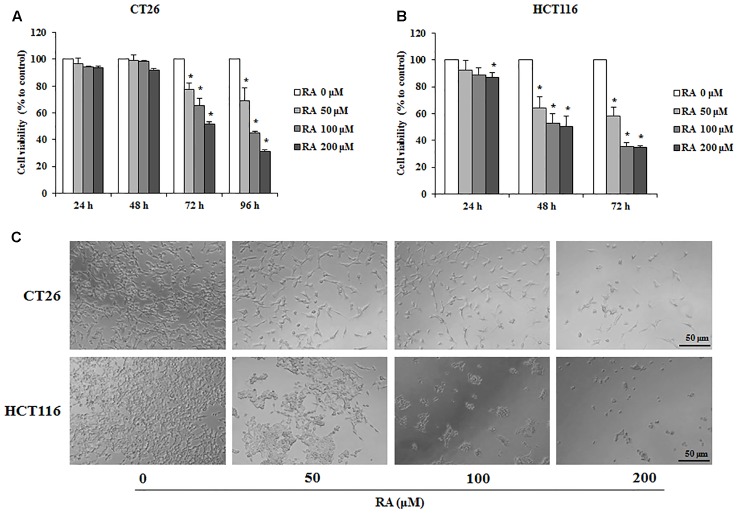
Rosmarinic acid (RA) exhibits an anti-proliferative effect on metastatic CRC cells. **(A,B)** The cell viability of RA-treated CT26 **(A)** and HCT116 **(B)** cells. The cells were seeded at a density of 3 × 10^3^ cells/well in 96-well microplates and treated with RA for 24, 48, 72, and 96 h. After treatment with RA, cell viability was measured by using the MTS assay. **(C)** The morphologies of CT26 and HCT116 cells treated with RA for 96 h and 72 h, respectively. The images were obtained by using a microscope (200× magnification). The results are expressed as the mean ± SD. ^∗^*p* < 0.05 indicates a significant difference from the untreated group.

To examine whether RA reduced cell survival in metastatic colon cancer cells, CT26 and HCT116 cells were used. The MTS assay was conducted to confirm the cytotoxic effect of RA. As shown in **Figures 1A,B**, RA treatment inhibited cell proliferation in a time- and concentration-dependent manner. The cytotoxic phenotypes were also observed under a microscope (**Figure 1C**). To exclude changes in factors due to decreased cell viability, we treated RA (50, 100, and 200 μM) for 48 h in CT26 cells and 24 h in HCT116 cells, which were not sufficient to affect cell viability.

### RA Induces Cell Cycle Arrest of G0/G1 Phase in CRC Cells

**FIGURE 2:**
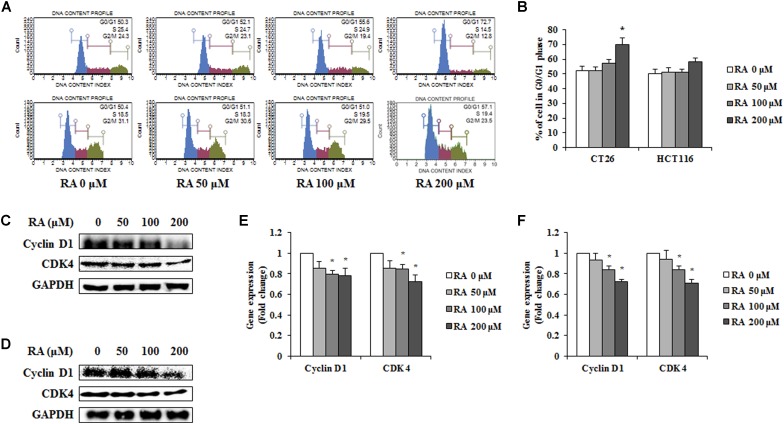
Rosmarinic acid (RA) induces G0/G1 phase arrest in CRC cells. **(A)** The cell cycle analysis of RA-treated CT26 cells for 48 h (upper part) and RA-treated HCT116 cells for 24 h (lower part) **(B)** The population of cells in each cell cycle phase in RA-treated CT26 and HCT116 cells. **(C,D)** The proteins expression of cyclin D1 and CDK4 in RA-treated CT26 cells for 48 h **(C)** and RA-treated HCT116 cells for 24 h **(D)**. **(E,F)** The mRNA expression of cyclin D1 and CDK4 in RA-treated CT26 cells for 48 h **(E)** and RA-treated HCT116 cells **(F)** for 24 h. GAPDH and β-actin were used as the endogenous control. The results are expressed as the mean ± SD. ^∗^*p* < 0.05 indicates a significant difference from the untreated group.

Cells have a self-replicating process known as the cell cycle. As cell cycle arrest suppresses cancer cell growth, it may offer an important strategy for cancer treatment (Bartek and Lukas, 2001). To confirm whether RA induced cell cycle arrest, CT26 and HCT116 cells were treated with RA and analyzed by flow cytometry. Compared with untreated CT26 cells, the percentage of cells in G0/G1 phase increased in RA-treated groups. Additionally, similar tendency was observed in HCT116 cells (**Figures 2A,B**). These results showed that RA-induced cell cycle arrest in the G0/G1 phase of metastatic CRC cells. To confirm the inhibitory mechanisms, we conducted Western blot and real-time RT-PCR. It was reported that cell cycle arrest in the G0/G1 phase was induced by a decrease in cyclin D-cdks (Pucci et al., 2000). As shown in **Figures 2C–F**, RA inhibited the protein levels and the mRNA expressions of cyclin D1 and CDK4. These results indicated that RA-mediated G0/G1 phase arrest resulted from decreased cyclin D1 and decreased CDK4.

### Effect of RA on Apoptosis of CRC Cells

**FIGURE 3:**
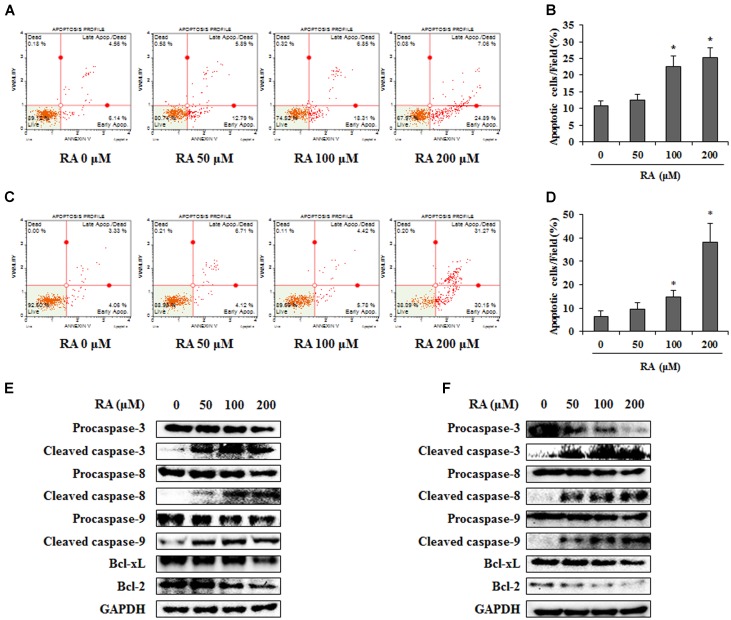
Rosmarinic acid (RA) induces apoptosis of CRC cells via the extrinsic and intrinsic apoptosis pathway. **(A)** CT26 cells were treated with the indicated concentrations of RA for 48 h and stained with annexin V and propidium iodide. **(B)** The quantification of apoptotic cells. The data indicate the percentage of apoptotic cells after RA treatment. **(C)** HCT116 cells were treated with the indicated concentrations of RA for 24 h and stained with annexin V and propidium iodide. **(D)** The quantification of apoptotic cells. The data indicate the percentage of apoptotic cells after RA treatment. **(E)** CT26 cells were treated with RA for 48 h and a Western blot was conducted to detect the expression of procaspase-3, cleaved caspase-3, procaspase-8, cleaved caspase-8, procaspase-9, cleaved caspase-9, PARP, Bcl-xL, and Bcl-2. **(F)** HCT116 cells were treated with RA for 24 h and Western blot was conducted. The expression of GAPDH used as an endogenous control. The results are expressed as the mean ± SD. ^∗^*p* < 0.05 indicates a significant difference from the untreated group.

As both apoptosis and cell cycle arrest are crucial pathways in the anti-cancer mechanism (Niknejad et al., 2016), we confirmed whether RA could induce apoptosis. To detect the apoptotic cells, we performed an annexin V assay after the treatment of RA (0, 50, 100, or 200 μM). As shown in **Figures 3A–D**, the percentage of apoptotic CT26 and HCT116 cells was significantly increased after treatment with 200 μM RA. To further investigate the mechanisms of apoptosis, Western blotting analysis was conducted. As shown in **Figures 3E,F**, the treatment of CT26 and HCT116 cells with RA reduced the protein levels of procaspase-9, Bcl-xL, and Bcl-2, which are related to the intrinsic apoptosis pathway (Ren et al., 2013). Procaspase-3 and procaspase-8, which are related to the extrinsic apoptosis pathway (Fatehchand et al., 2017), were reduced by RA. Continually, cleavage of caspase-3, -8, and -9 were increased by RA treatment in CT26 and HCT116 cells. These results suggested that RA induced apoptosis via the intrinsic and extrinsic pathways.

### RA Regulated EMT of CRC Cells

**FIGURE 4:**
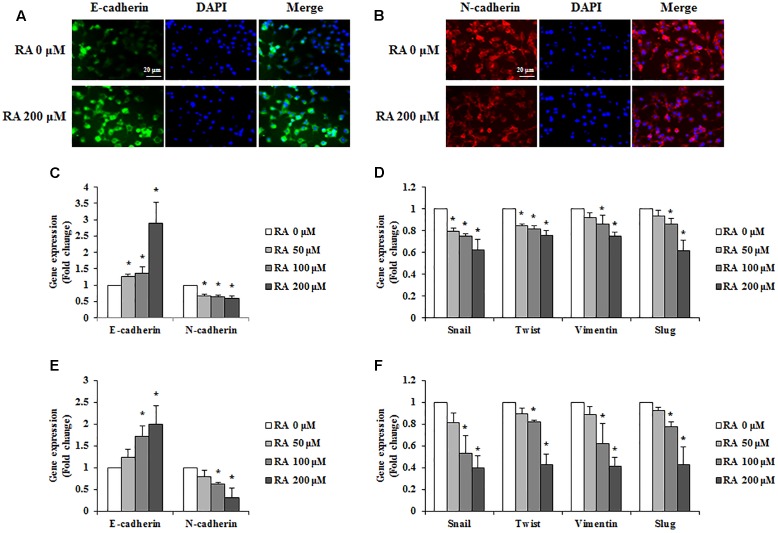
Rosmarinic acid (RA) regulates the EMT of CRC cells. **(A,B)** Representative immunofluorescence images of E-cadherin **(A)** and N-cadherin **(B)** in RA-treated CT26 cells for 48 h. The nuclei were stained with DAPI (blue) and the equivalent phase contrast images were taken by fluorescent microscope. **(C,E)** The mRNA expression of E-cadherin and N-cadherin in RA-treated CT26 cells **(C)** and HCT116 cells **(E)**. **(D,F)** The mRNA expression of snail, twist, vimentin, and slug in RA-treated CT26 cells **(D)** and HCT116 cells **(F)**. GAPDH and β-actin were used as the endogenous control. The results are expressed as the mean ± SD. ^∗^*p* < 0.05 indicates a significant difference from the untreated group.

For cancer cells to metastasize, the epithelial phenotype has to change to a mesenchymal phenotype; this phenomenon is known as EMT (Sowa et al., 2015). To investigate whether RA could regulate the EMT, we conducted immunofluorescence staining to detect the expression of E-cadherin and N-cadherin. As shown in **Figures 4A,B**, the expression of E-cadherin, an epithelial marker, was increased, and N-cadherin, a mesenchymal marker, was inhibited in CT26 cells treated with RA. Additionally, the real-time RT-PCR data were consistent with the results of immunofluorescence staining (**Figure 4C**). Other EMT-related mesenchymal markers, including snail, twist, vimentin, and slug, were inhibited by treatment with RA (**Figure 4D**). In HCT116 cells, RA also regulated expressions of EMT-related makers to inhibit EMT process (**Figures 4E,F**).

### RA Disrupts the Migration, Invasion, and Adhesion Abilities of CRC Cells

**FIGURE 5:**
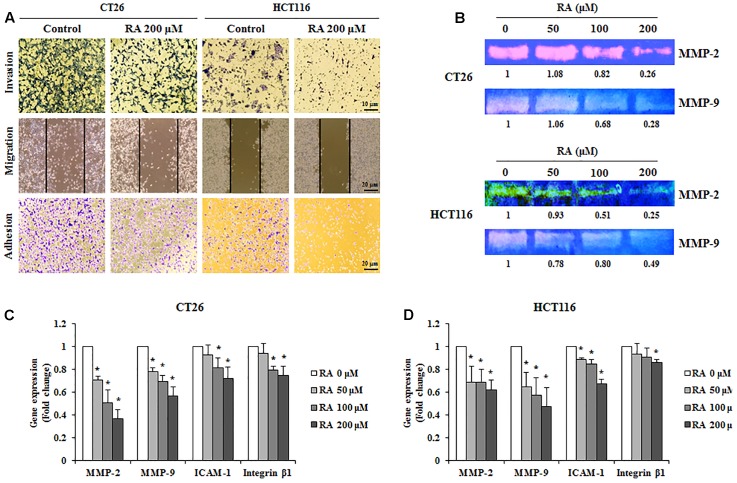
Rosmarinic acid (RA) reduces the migration, invasion, and adhesion of CRC cells and inhibits the expression of related factors. **(A)** Invasion assay (upper), wound healing assay (middle), and adhesion assay (lower) of RA-treated CT26 and HCT116 cells. After treatment of RA for 48 h in CT26 cells and 24 h in HCT116 cells, the images were collected by using a microscope (200×, 100×, and 100×, respectively). **(B)** The activities of MMP-2 and MMP-9 were determined by gelatin zymography in CT26 cells treated with RA for 48 h and HCT116 cells treated with RA for 24 h. **(C,D)** The mRNA expressions of MMP-2, MMP-9, ICAM-1, and Integrin β1 in CT26 cells treated with RA for 48 h **(C)**, and HCT116 cells treated with RA for 24 h **(D)**. The results are expressed as the mean ± SD. ^∗^*p* < 0.05 indicates a significant difference from the untreated group.

For cancer cells to metastasize, the cells of primary tumors invade the ECM and migrate to the secondary site. During this process, circulating cells, which survive from the immune system, eventually get attached to the secondary site (Reymond et al., 2013). Therefore, the inhibition of invasion, metastasis, and adhesion could be an important strategy for the prevention of metastasis. As shown in **Figure 5A**, the treatment of CRC cells with RA inhibited invasion, migration, and adhesion. We confirmed the levels of MMP-2 and MMP-9, which promote ECM degradation and induce invasion and migration. To analyze the expression and activities of MMP-2 and MMP-9, real-time RT-PCR and gelatin zymography were conducted. As shown in **Figures 5B–D**, RA reduced the expression and activities of MMP-2 and MMP-9 in CT26 cells. Moreover, the adhesion-related factors, ICAM-1 and integrin β1, were decreased by RA treatment (**Figures 5C,D**). These results suggested that RA could inhibit invasion, migration, and invasion.

### RA Inhibits EMT, Invasion, and Migration via AMPK Phosphorylation in CRC Cells

**FIGURE 6:**
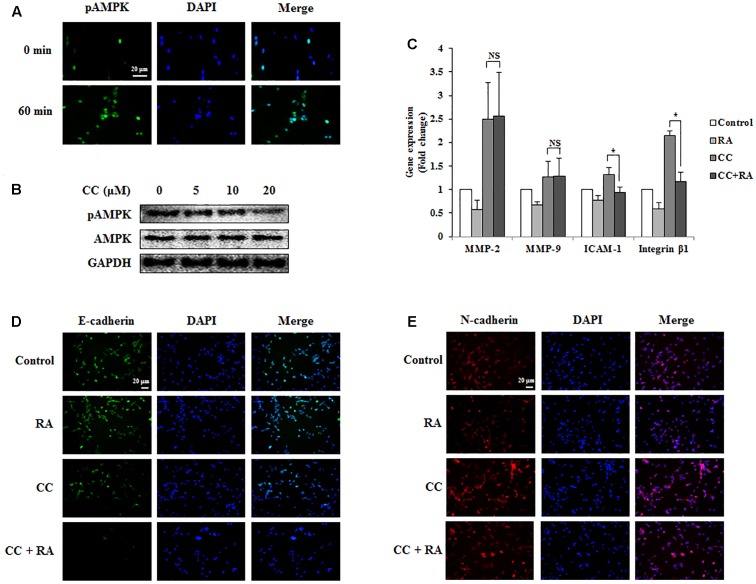
Rosmarinic acid (RA) inhibits the EMT, migration, and invasion through AMPK activation in CT26 cells. **(A)** Representative immunofluorescence images of phospho-AMPK. The nuclei were stained with DAPI (blue) and equivalent phase contrast images were obtained by fluorescence microscopy. Scale bar = 20 μm. **(B)** CT26 cells were treated with CC for 4 h and detected phosphorylation levels of AMPK. **(C)** The mRNA expressions of MMP-2, MMP-9, ICAM-1, and integrin β1. GAPDH was used as the endogenous control. **(D,E)** Representative immunofluorescence images of E-cadherin **(D)** and N-cadherin **(E)**. CC (20 μM) was pre-treated for 4 h and then CT26 cells were treated with RA for 48 h. The nuclei were stained with DAPI (blue) and equivalent phase contrast images were obtained by fluorescence microscopy. Scale bar = 20 μm. The results are expressed as the mean ± SD. ^∗^*p* < 0.05 indicates significant differences from the CC-treated group. ^NS^*p* > 0.05 indicates no significant differences from the CC-treated group.

**FIGURE 7:**
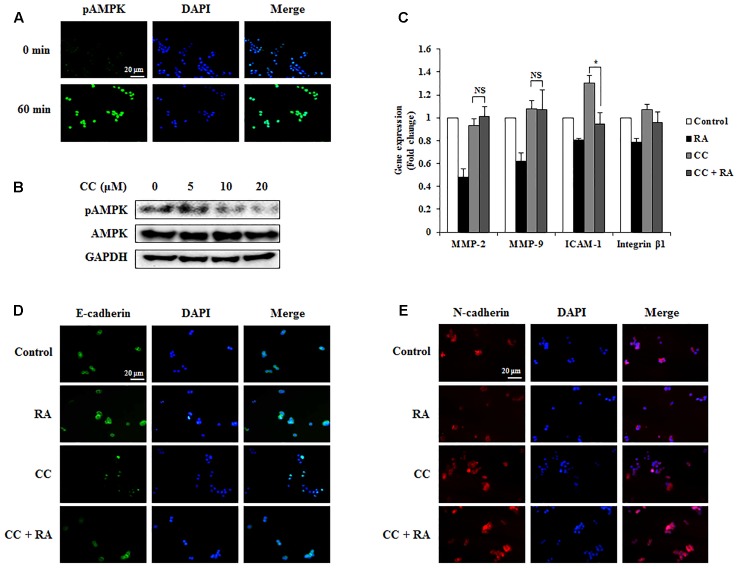
Rosmarinic acid (RA) inhibits the EMT, migration, and invasion through AMPK activation in HCT116 cells. **(A)** Representative immunofluorescence images of phospho-AMPK. The nuclei were stained with DAPI (blue) and equivalent phase contrast images were obtained by fluorescence microscopy. Scale bar = 20 μm. **(B)** HCT116 cells were treated with CC for 4 h and detected phosphorylation levels of AMPK. **(C)** The mRNA expression of MMP-2, MMP-9, ICAM-1, and integrin β1. GAPDH and β-actin were used as the endogenous control. **(D,E)** Representative immunofluorescence images of E-cadherin **(D)** and N-cadherin **(E)**. CC (20 μM) was pre-treated for 4 h and then HCT116 cells were treated with RA for 24 h. The nuclei were stained with DAPI (blue) and equivalent phase contrast images were obtained by fluorescence microscopy. Scale bar = 20 μm. The results are expressed as the mean ± SD. ^∗^*p* < 0.05 indicates significant differences from the CC-treated group. ^NS^*p* > 0.05 indicates no significant differences from the CC-treated group.

The phosphorylation of AMPK was associated with better prognosis and survival rate (Baba et al., 2010; Esfahanian et al., 2012) and with inhibition of EMT in lung and breast cancers (Chou et al., 2014; Qu et al., 2014; Lin et al., 2015). Several studies have reported that the activation of AMPK inhibited the expression of MMP-2 and MMP-9, which are crucial factors related to invasion and migration (Morizane et al., 2011). It was also reported that AMPK could regulate ICAM-1 and integrin β1 (Ha et al., 2015). First, we confirmed whether RA induced the phosphorylation of AMPK in CRC cells. As shown in **Figures 6A**, **7A**, RA treatment induced AMPK phosphorylation. Next, to elucidate the correlation between AMPK phosphorylation and the anti-metastatic effect of RA, we pre-incubated the cells with CC, a potent AMPK inhibitor, and then treated RA for 48 h in CT26 cells and 24 h in HCT116 cells. Phosphorylation of AMPK was sufficiently blocked by CC treatment (**Figures 6B**, **7B**). CC pretreatment disrupted the inhibitory effect of RA on the expressions of MMP-2 and MMP-9 in both cell lines. However, the expressions of the adhesion molecules ICAM-1 and integrin β1 were reduced by RA, despite the blocking of AMPK activation (**Figures 6C**, **7C**). The expression of the EMT markers E-cadherin and N-cadherin was not regulated after CC pretreatment and the subsequent RA treatment in CT26 cells (**Figures 6D,E**). In addition, similar result was shown in HCT116 cells (**Figures 7D,E**). These results indicated that the anti-metastatic effects of RA on the EMT, invasion, and migration a result of AMPK phosphorylation.

### RA Reduces Lung Metastasis of CRC Cells via Activation of AMPK in an Experimental Metastasis Model

**FIGURE 8:**
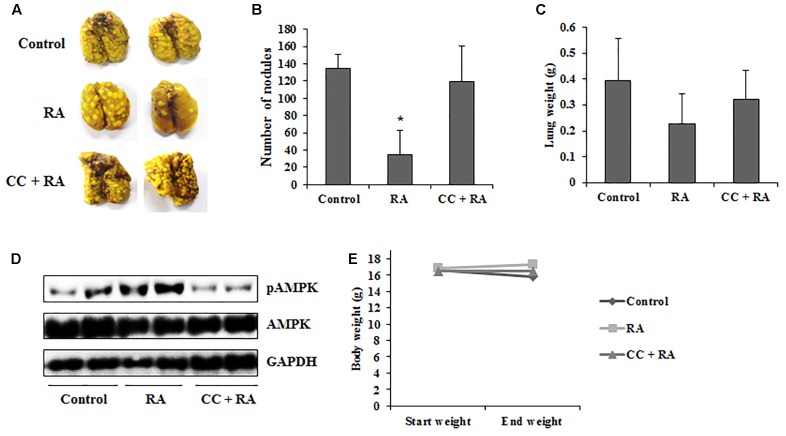
Rosmarinic acid (RA) inhibits lung metastasis of CRC cells by activating AMPK. BALB/c mice were intravenously transplanted with 1 × 10^5^ cells of CT26 cells into the tail vein. Then, the mice were divided into three groups (*n* = 7). RA (100 mg/kg) was administered by oral gavage once a day until sacrifice. CC (10 mg/kg) was injected every 3 days. **(A)** Lungs were stained with Bouin’s solution to compare the pattern of tumor nodule formation. **(B,C)** The number of tumor nodules **(B)** and lung weights **(C)**. **(D)** The protein levels of phospho-AMPK were detected in lung tissues. **(E)** Body weights of experiment groups. The results are expressed as the mean ± SD. ^∗^*p* < 0.05 indicates significant differences from the control group.

Our study also investigated that whether RA could ameliorate lung metastasis by activating AMPK. As shown in **Figures 8A–C**, the numbers of pulmonary tumor nodules and lung weights were decreased by oral administration of RA. Moreover, these anti-metastatic effects of RA were ameliorated by CC treatment. Compared to the control group, phosphorylation of AMPK was increased in RA-treated group, whereas it was blocked in CC + RA group (**Figure 8D**). Body weight, an indicator of toxicity, was not reduced by RA administration (**Figure 8E**). Furthermore, RA did not show the hepatotoxicity and nephrotoxicity (**Table 3**). These results indicated that RA could inhibit the lung metastasis of CRC cells through AMPK activation.

**Table 3:** Liver and kidney parameters on serum levels in experimental mice.

	AST (IU/L)	ALT (IU/L)	Creatinine (mg/dL)	BUN (mg/dL)
Control	183.00 ± 36.54	39.00 ± 11.20	0.10 ± 0.02	22.60 ± 1.82
RA	119.40 ± 36.85	36.40 ± 12.74	0.10 ± 0.03	22.74 ± 3.30
RA + CC	141.20 ± 51.38	36.60 ± 6.02	0.12 ± 0.04	23.50 ± 3.70


## Discussion

The main treatments for cancer currently comprise surgery, chemotherapy, and radiation therapy. Although surgery is the most reliable method, it is suitable only for solid tumors. Furthermore, chemotherapy, such as the administration of chemical drugs and radiotherapy, is accompanied by considerable side effects. In severe cases, the cancer treatment itself leads to mortality (Joshi et al., 2009). For these reasons, the bioactive compounds in natural products, oriental medicine, and spices have been studied as substitutes for cancer drugs and to reduce the severity of the side effects (Li et al., 2011).

Rosmarinic acid, which is commonly abundant in Boraginaceae and Lamiaceae, is not only easy to obtain, but can also be ingested as tea or food (Petersen and Simmonds, 2003). It is well known for its anti-inflammatory effects and has been used for the prevention of cancer in folk medicine (Elansary and Mahmoud, 2015). Xu et al. reported that RA inhibits invasion via the oxidation–reduction pathway in LS174T cells, whereas the specific molecular inhibitory mechanism of RA remains unclear (Xu et al., 2010b). Furthermore, RA could improve the efficiency of radiation therapy by exerting a radiosensitizing effect on cancer cells (Alcaraz et al., 2014). Various studies focused on AMPK, which is involved in various roles in a wide range of tissues such as liver, muscle and adipose tissues, as a new therapeutic target of cancer (Hardie et al., 2003). In addition, many evidences showed that the AMPK pathway controls the processes of cancer cell migration and invasion (Li N. et al., 2015). However, there was no study on the effects of RA on the CRC via AMPK pathway. Therefore, the study of the AMPK related mechanism will allow RA to be used in an adjuvant chemotherapy or chemotherapy. In the present study, we first confirmed the anti-proliferative effect of RA on the metastatic murine and human CRC cell line, CT26 and HCT116. As expected, treatment with RA inhibited the proliferation of CRC (**Figure 1**). As the anti-proliferative effect was closely related to cell cycle arrest and apoptosis (Niknejad et al., 2016), we confirmed the changes in the cell cycle of CT26 cells after treatment with RA. RA induced cell cycle arrest through an increase in the percentage of cells in the G0/G1 phase (**Figures 2A,B**). To proceed from the G0/G1 phase to the S phase, cyclin D1 activated CDK4. As shown in **Figures 2C–F**, the treatment with RA decreased the protein and the mRNA expressions of cyclin D1 and CDK4. Thus, our data demonstrated that RA induced G0/G1 phase arrest through the regulation of the expression of cyclin D1 and CDK4.

Apoptosis, which is classified into the extrinsic and intrinsic pathways, is deregulated in cancer cells. The induction of apoptosis is a therapeutic strategy for the treatment of cancer (Hassan et al., 2014). In this study, we confirmed that RA induced apoptosis of CRC cells (**Figures 3A–D**). To investigate the related mechanisms, RA-regulated protein levels were analyzed. The extrinsic apoptosis pathway is induced by the tumor necrosis factor family. Once this pathway is activated, procaspase-8 was cleaved to caspase-8 and then procaspase-3 was also cleaved to caspase-3, which leads to cell death (Lowe and Lin, 2000; Safa, 2012). As shown in **Figures 3E,F**, RA induced the cleavage of caspase-3, caspase-8, and caspase-9. In the intrinsic apoptosis pathway, the anti-apoptotic proteins Bcl-2 and Bcl-xL exert self-inhibitory action and emit cytochrome c. Cytochrome c induced apoptosis and converted procaspase-9 to active caspase-9 (Deveraux et al., 1998; Verhagen et al., 2000). Our data showed that RA downregulated procaspase-9, Bcl-xL, and Bcl-2 in CRC cells (**Figures 3E,F**). These results indicated that RA induced apoptosis through the extrinsic and intrinsic apoptosis pathways.

Cancer cells that become detached from the primary tumor circulate, reach distant organs, and then form a secondary tumor. EMT causes cancer cells to lose cell-to-cell contacts through an adoption of mesenchymal features, which are required for detachment from the primary tumor (Tian et al., 2016). During this process, epithelial markers, such as E-cadherin, are downregulated, whereas the mesenchymal markers such as N-cadherin, snail, twist, vimentin and slug are upregulated. Therefore, the inhibition of EMT is crucial for the prevention of metastasis. To confirm the expression of E-cadherin and N-cadherin, we conducted immunofluorescence staining. The treatment of CRC cells with RA increased E-cadherin and decreased N-cadherin (**Figures 4A,B**). Similar to the immunofluorescence staining results, the mRNA levels of E-cadherin and N-cadherin were regulated by treatment with RA (**Figure 4C**). Furthermore, mesenchymal markers, including snail, twist, vimentin and slug, were inhibited by treatment with RA (**Figure 4D**). In addition, EMT-related markers were also regulated by RA treatment in HCT116 cells (**Figures 4E,F**). These results suggested that RA inhibited the EMT of metastatic CRC cells.

After the EMT occurred, the cancer cells underwent migration, invasion, and adhesion (Ondroušková et al., 2016). Detached cancer cells migrate and invade the extracellular matrix, which is destroyed by MMPs (Huang et al., 2015). Therefore, the inhibition of migration and invasion through the reduction of the expression and activity of MMPs would be a sound strategy for the inhibition of metastasis. RA treatment suppressed invasion (**Figure 5A**, upper part) and migration (**Figure 5A**, middle part). Furthermore, the expression and activity of MMP-2 and MMP-9 were decreased by treatment with RA (**Figures 5B–D**). The cancer cells that survive in the bloodstream become attached to the secondary site and the adhesion molecules, such as ICAM-1 and integrin β1, affect the adhesion process (Weiss and Ward, 1983). Similar to previous results, the adhesion of CT26 and HCT116 cells were suppressed (**Figure 5A**, lower part) and ICAM-1 and integrin β1, which are adhesion molecules, were also suppressed by treatment with RA (**Figures 5C,D**).

Next, to elucidate the specific mechanisms of the inhibitory effects of RA, we conducted additional experiments to investigate whether RA could activate AMPK. AMPK was reported to regulate the balance of cell growth, energy homeostasis, type II diabetes, obesity, metabolic disease, and cancer (Kang et al., 2013; Monteverde et al., 2015). Recently, several studies demonstrated that the AMPK pathway suppressed invasion and migration through a reduction in MMPs (Petursson et al., 2013; Li N. et al., 2015). Further, AMPK activation inhibited EMT through the regulation of EMT-related makers and abolished the attachment of cancer cells through the inhibition of ICAM and integrin (Chen et al., 2011; Lee et al., 2013). In addition, the p53 tumor suppressor gene was directly phosphorylated by AMPK. Therefore, the regulation of AMPK signaling may be a key factor for the treatment of cancer genesis and metastasis (Park et al., 2015). It has been reported that RA could activate AMPK in skeletal muscle (Jayanthy et al., 2017). Thus, we first confirmed whether RA could activate AMPK in CRC cells. As shown in **Figures 6A**, **7A**, RA increased AMPK phosphorylation, and when the activation of AMPK was inhibited by CC pre-treatment, RA was no longer able to regulate the expression of E-cadherin and N-cadherin (**Figures 6D,E**, **7D,E**). Furthermore, MMP-2 and MMP-9 did not appear to be regulated by RA treatment after the inhibition of AMPK (**Figures 6C**, **7C**). Although it appeared that the metastatic traits were exacerbated by pre-treatment with CC, RA could not regulate the EMT and MMPs in when AMPK was inactive. However, RA suppressed adhesion of CRC cells during AMPK inactivation by CC treatment (**Figures 6C**, **7C**). Several studies have reported that NLRC3, which is different pathway from AMPK, inhibited activation of PI3K/AKT/mTOR axis thereby inhibiting cell proliferation and inducing apoptosis of CRC cells (Karki et al., 2016, 2017). To confirm further whether RA could regulate progression of CRC through other signaling pathway, we also examined gene expression level of NLRC3. However, RA did not regulate the expression of NLRC3 (Supplementary Figure S1). These results suggested that the inhibitory effects of RA on EMT, migration, and invasion ability might be controlled by AMPK activation.

The spontaneous metastasis model is more similar to the clinical cases. However, it took a long time and showed low successful rate (Khanna and Hunter, 2005). For these reasons, we selected an experimental metastasis *in vivo* model to confirm whether RA could inhibit the metastasis via AMPK pathway. As shown in **Figure 8**, RA significantly decreased the number of metastatic tumor nodules in lungs by activating AMPK. Although there was no statistical significance, the increase in lung weight due to tumor nodules was inhibited. However, AMPK blocking by CC treatment inhibited the anti-metastatic effects of RA.

Collectively, this study elucidated that RA induced cell death in metastatic CRC cells and inhibited their metastatic properties. RA induced cycle arrest in the G0/G1 phase and apoptosis in CRC cells. It also inhibited metastatic abilities of CRC cells including EMT, migration, and invasion through activation of AMPK. The *in vivo* experiment proved that RA could reduce lung metastasis by activating AMPK. Based on our results, RA may be a natural therapeutic drug for the treatment of colon cancer and metastasis.

## Author Contributions

Y-HH, J-YK, and S-HH designed the experiments and wrote the manuscript. Y-HH performed the experiments and analyzed the data. S-HH supervised all the experiments and analyses.

## Conflict of Interest Statement

The authors declare that the research was conducted in the absence of any commercial or financial relationships that could be construed as a potential conflict of interest.
