# Supplementary material for: Rosmarinic Acid Activates AMPK to Inhibit Metastasis of Colorectal Cancer
Source: Front Pharmacol. 2018 Feb 5;9:68. doi: 10.3389/fphar.2018.00068 (PMC5807338; doi:10.3389/fphar.2018.00068)
Supplement: Supplementary file 1 [file Data_Sheet_1.DOCX]

Supplementary Material

Rosmarinic acid activates AMPK to inhibit metastasis of colorectal cancer

***Yo-Han Han, Ji-Ye Kee and Seung-Heon Hong***

*** Correspondence:** Seung-Heon Hong: jooklim@wku.ac.kr

# Supplementary Figures and Tables

## Supplementary Figure

**Supplementary Figure S1**


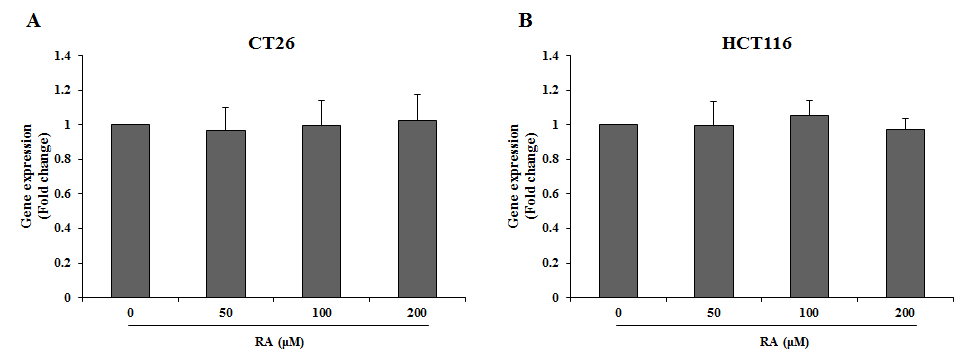


**Supplementary Figure S1.** The mRNA expression of NLRC3 in RA-treated CT26 cells (A) and HCT116 cells (B). GAPDH and β-actin were used as the endogenous control. Primer sequences were presented in Table 1 and Table 2. The results are expressed as the mean ± SD.
